# Supplementary material for: The interactome of intact mitochondria by cross-linking mass spectrometry provides evidence for coexisting respiratory supercomplexes
Source: Mol Cell Proteomics. 2017 Dec 8;17(2):216–32. doi: 10.1074/mcp.RA117.000470 (PMC5795388; doi:10.1074/mcp.RA117.000470)
Supplement: Supplemental Data [file supp_RA117.000470_133922_0_supp_23978_fzffwf.zip › spectra_annotation/mito_spectra_annotation/355-1-15-1-21-1.pdf]

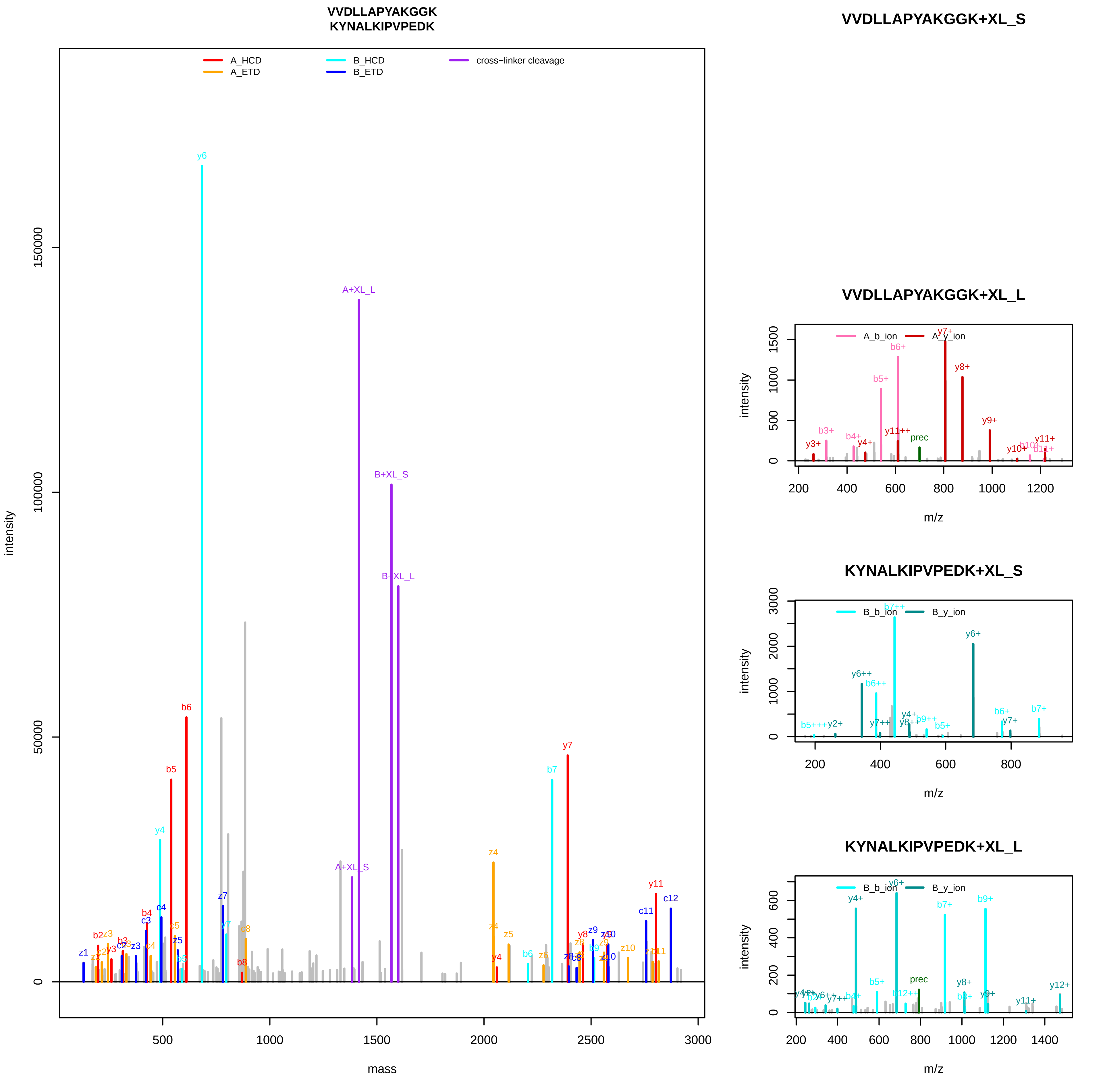
VVDLLAPYAKGGK
KYNALKIPVPEDK
A_HCD
A_ETD
B_HCD
B_ETD
cross−linker cleavage
intensity
mass
0
50000
100000
150000
500
1000
1500
2000
2500
3000
y6
A+XL_L
B+XL_S
B+XL_L
A+XL_S
b6
b5
y7
b7
y4
z7
z4
y11
c12
c11
VVDLLAPYAKGGK+XL_S
VVDLLAPYAKGGK+XL_L
A_b_ion
A_y_ion
m/z
y7+
b6+
y8+
b5+
y9+
prec
y11++
b3+
y3+
b4+
y4+
y10+
y11+
KYNALKIPVPEDK+XL_S
B_b_ion
B_y_ion
b7++
y6+
y6++
b6++
b7+
b6+
y7+
KYNALKIPVPEDK+XL_L
y4+
b7+
b9+
b5+
y8+
y9+
y12+
y11+
